# Classifying Unstructured Text in Electronic Health Records for Mental Health Prediction Models: Large Language Model Evaluation Study

**DOI:** 10.2196/65454

**Published:** 2025-01-21

**Authors:** Nicholas C Cardamone, Mark Olfson, Timothy Schmutte, Lyle Ungar, Tony Liu, Sara W Cullen, Nathaniel J Williams, Steven C Marcus

**Affiliations:** 1 Department of Psychiatry, Perelman School of Medicine, University of Pennsylvania, Philadelphia, PA, United States; 2 Department of Psychiatry, the New York State Psychiatric Institute, New York, NY, United States; 3 Department of Psychiatry, Yale School of Medicine, New Haven, CT, United States; 4 Computer and Information Science, University of Pennsylvania, Philadelphia, PA, United States; 5 School of Social Policy & Practice, University of Pennsylvania, Philadelphia, PA, United States; 6 School of Social Work, Boise State University, Boise, ID, United States

**Keywords:** artificial intelligence, AI, machine learning, ML, natural language processing, NLP, large language model, LLM, ChatGPT, predictive modeling, mental health, health informatics, electronic health record, EHR, EHR system, text, dataset, mental health disorder, emergency department, physical health

## Abstract

**Background:**

Prediction models have demonstrated a range of applications across medicine, including using electronic health record (EHR) data to identify hospital readmission and mortality risk. Large language models (LLMs) can transform unstructured EHR text into structured features, which can then be integrated into statistical prediction models, ensuring that the results are both clinically meaningful and interpretable.

**Objective:**

This study aims to compare the classification decisions made by clinical experts with those generated by a state-of-the-art LLM, using terms extracted from a large EHR data set of individuals with mental health disorders seen in emergency departments (EDs).

**Methods:**

Using a dataset from the EHR systems of more than 50 health care provider organizations in the United States from 2016 to 2021, we extracted all clinical terms that appeared in at least 1000 records of individuals admitted to the ED for a mental health–related problem from a source population of over 6 million ED episodes. Two experienced mental health clinicians (one medically trained psychiatrist and one clinical psychologist) reached consensus on the classification of EHR terms and diagnostic codes into categories. We evaluated an LLM’s agreement with clinical judgment across three classification tasks as follows: (1) classify terms into “mental health” or “physical health”, (2) classify mental health terms into 1 of 42 prespecified categories, and (3) classify physical health terms into 1 of 19 prespecified broad categories.

**Results:**

There was high agreement between the LLM and clinical experts when categorizing 4553 terms as “mental health” or “physical health” (κ=0.77, 95% CI 0.75-0.80). However, there was still considerable variability in LLM-clinician agreement on the classification of mental health terms (κ=0.62, 95% CI 0.59‐0.66) and physical health terms (κ=0.69, 95% CI 0.67‐0.70).

**Conclusions:**

The LLM displayed high agreement with clinical experts when classifying EHR terms into certain mental health or physical health term categories. However, agreement with clinical experts varied considerably within both sets of mental and physical health term categories. Importantly, the use of LLMs presents an alternative to manual human coding, presenting great potential to create interpretable features for prediction models.

## Introduction

Recent advances in health informatics have led to the development of machine learning models that are trained on data from electronic health records (EHRs). These models have proven to be effective across a range of health domains, including predicting the spread of disease [1], hospital readmission rates [2], and suicide risk [3,4]. Predictive models have been implemented in EHR systems to identify high-risk patients and alert clinicians to critical health events [5].

EHR systems are filled with unstructured text data, including clinical notes and discharge summaries, which are not easily categorized into clinically interpretable groupings for use in predictive models. Although the use of this data can greatly enhance prediction model performance and the interpretability of decision-support tools [6,7], the coding process is labor intensive and requires expert consultation and extensive training [8]. These challenges hinder the development and scalability of clinical prediction models that incorporate unstructured EHR data [9,10].

Large language models (LLMs), like OpenAI’s GPT models, can streamline the classification and coding of unstructured EHR text due to their massive training data sets and advanced text processing [11,12]. LLMs have been used to categorize unstructured text from EHR systems [13], assist with qualitative analysis [14,15], and perform deductive coding with and without context [16]. Preliminary evidence shows that LLMs outperform crowd workers in annotation of health texts [17,18].

The reliability of LLMs in replicating clinical judgment for coding classification tasks in mental health remains uncertain, particularly given the inherent complexities of mental health disorders [19,20]. Prior research highlights that while LLMs can process large volumes of text, their ability to discern subtle differences in clinical presentations, such as differentiating between comorbid conditions like depression and anxiety, is still unproven. This challenge is exacerbated by the frequent overlap of symptoms across diagnoses, which complicates classification efforts [21]. Patients with mental health disorders may present with unique clinical characteristics that challenge an LLM’s ability to accurately identify and code physical and mental health symptoms [11,22,23].

We used a large EHR data set of individuals admitted to the emergency department (ED) for a mental health disorder, to assess the ability of a state-of-the-art LLM to classify EHR terms into categories defined by experienced mental health clinicians. We assessed the extent to which a LLM replicates clinical judgment and the practicality of using a LLM to assist in creating clinically interpretable features for prediction models.

## Methods

### Data

We extracted de-identified EHR data from the Optum Labs Data Warehouse, a longitudinal, real-world data asset, from >50 US healthcare provider organizations that encompass >700 hospitals. We included individuals aged ≥10 years who were admitted to the ED from 2016 to 2021 and had an International Classification of Disease-9 or -10 code for a mental health diagnosis, suicidal ideation, or self-harm, resulting in approximately 6.2 million unique patient episodes. A natural language processing (NLP) algorithm integrated into the Optum Labs Data Warehouse extracted from unstructured free-text fields in the EHR, clinical terms for signs, symptoms, and diseases based on the National Library of Medicine’s Unified Medical Language System dictionary. We identified physical and mental health terms that appeared in at least 1000 unique patient episodes.

### Coding

A board-certified psychiatrist and licensed clinical psychologist categorized each EHR term into 1 of 61 categories including 42 mental health-related categories and 19 physical health-related categories which were generated from the Clinical Classifications Software Refined [24] and the International Classification of Disease-10 diagnosis coding system, respectively. Coding each EHR term involved: (1) initial classification by 1 clinician coder, (2) a review of all coding decisions by a second clinician coder with suggestions for revisions; (3) a final consensus reconciliation involving both coders. The coding of physical health terms was supported by an LLM, which suggested coding decisions that were refined and reconciled (5% of terms required reconciliation) by the 2 clinician coders. All study procedures were approved by the Institutional Review Board of University of Pennsylvania.

### Classification Tasks

We used the Python module “openai” [25] to run the GPT-4 LLM in a Python environment. We used OpenAI’s most sophisticated GPT-4 that was then publicly available (“gpt-4-turbo-2024-04-09”) and set model parameters to maximize output consistency (eg, temperature=0).

We prompted the model with 3 “zero-shot” classification tasks, wherein the model is provided codes without examples: (1) classify all (n=4553) EHR terms as either “mental health” or “physical health,” (2) classify each of the (n=846) mental health terms into 1 of the 42 mental health categories, and (3) classify each of the (n=3707) physical health terms into 1 of the 19 physical health categories. The prompt described the task, listed the possible categories, and provided the EHR terms. The model then confirmed that the predicted category was among the list of possible categories. For full reproducibility, the complete prompt provided to the model, including the task description and category list, is detailed in Multimedia Appendix 1. In task 2, the model was given an unstructured clinical term from an EHR such as “depressive symptoms.” Then, the prompt described the classification task and provided the following list of possible mental health categories (eg, “depression,” “anxiety,” “eating disorder symptoms,” and “substance use”). The process was repeated for all 846 mental health terms, and similarly for the 3707 physical health terms in task 3.

### Performance Metrics

We compared GPT-4’s predicted categories with the categories determined by clinical judgment using the Python library scikit-learn “metrics” module [26]. For each task, we report the overall Cohen κ and weighted average of precision, recall, and *F*_1_-score, accounting for label imbalance. We computed 95% CIs for Cohen κ, precision, recall, and *F*_1_-score using a bootstrap procedure with 1000 resamples [27].

### Ethical Considerations

Ethical approval (IRB Protocol #848806) for this study was waived by the University of Pennsylvania Institutional Review Board via 45 CFR 46.104, category 4.

## Results

### Overview

EHR terms (n=4553) were categorized by GPT as “mental health” or “physical health.” Overall, classification performance was strong with κ of 0.77 (95% CI 0.75-0.80), precision of 0.93 (95% CI 0.92‐0.94), recall of 0.93 (95% CI 0.92‐0.94), and *F*_1_-score of 0.93 (95% CI 0.92‐0.94). The GPT-4 classified 18.3% (n=833) of the EHR terms as “mental health” and 81.7% (n=3720) as “physical health” (Table 1). The clinician coders and model disagreed on the categorization of 164 (19.7%) mental health terms (eg, “gunshot wound,” “chronic fatigue syndrome,” and “IV drug use”) and 149 (4%) physical health terms (eg, “activity issues,” “lethargic,” and “food issues”).

**Table 1.** Recall, *F*_1_-score, and total mentions among terms in the data set across health domains.

Health domain (n)	Recall (95% CI)^a^	*F*_1_-score (95% CI)	Total mentions in data set (thousands)
Physical health (n=3707)	0.96 (0.95‐0.97)	0.96 (0.95‐0.96)	255,573
Mental health (n=846)	0.81 (0.78‐0.83)	0.81 (0.79‐0.83)	85,081

a Recall indicates the proportion of terms in a clinician-coded category that were classified by the model as belonging to that category.

### Mental Health

Mental health terms (n=846) were classified into 42 categories with κ of 0.62 (95% CI 0.59-0.66), precision of 0.71 (95% CI 0.68‐0.74), recall of 0.64 (95% CI 0.61‐0.68), and *F*_1_-score of 0.65 (95% CI 0.62‐0.69). Table 2 includes category-wise recall, *F*_1_-score, and a set of the most frequent categories into which terms from the true category were misclassified (Multimedia Appendix 2).

**Table 2.** Mental health term categories: recall, *F*_1_-score, total mentions in the dataset, and most common misclassification (in descending order of recall). Categories with <5 terms were excluded.

Term category (n)	Recall (95% CI)^a^	*F*_1_-score (95% CI)	Total mentions in dataset (thousands)	Misclassifications (n)
Eating disorder or symptoms (n=16)	1 (0.81‐1)	0.91 (0.80‐1)	582	None
Living situation (n=11)	1 (0.74‐1)	1 (1‐1)	1259	None
ADHD^b^ spectrum (n=11)	1 (0.74‐1)	0.73 (0.52-0.88)	810	None
OCD^c^ symptoms or disorder (n=10)	1 (0.72‐1)	0.87 (0.67‐1)	207	None
Somatization symptoms (n=6)	1 (0.61‐1)	0.86 (0.57‐1)	62	None
Neurocognitive disorders (n=20)	0.95 (0.76‐0.99)	0.62 (0.47‐0.75)	1225	Neurocognitive symptoms (n=1)
Sleep wake symptoms or disorder (n=37)	0.95 (0.82‐0.99)	0.86 (0.78‐0.94)	1833	Miscellaneous psychiatric symptoms (n=1) and depressive symptoms (n=1)
Substance-related symptoms or disorder (n=90)	0.92 (0.85‐0.96)	0.95 (0.91‐0.98)	8783	Neurocognitive disorders (n=4), neurocognitive symptoms (n=1), and psychotic symptoms or disorder (n=1)
Abusive behavior (n=26)	0.89 (0.71‐0.96)	0.84 (0.71‐0.93)	3053	Aggressive symptoms (n=1), miscellaneous psychiatric symptoms (n=1), and personality disorder (n=1)
Unipolar depressive disorder (n=8)	0.88 (0.53‐0.98)	0.78 (0.50‐0.96)	944	Mood disorder (n=1)
Autism spectrum disorder (n=7)	0.86 (0.49‐0.97)	0.71 (0.38‐0.92)	132	Mood disorder (n=1)
Impulsive behavior (n=6)	0.83 (0.44‐0.97)	0.83 (0.50‐1)	414	Aggressive symptoms (n=1)
Personality disorder (n=5)	0.80 (0.38‐0.96)	0.47 (0.11‐0.73)	158	OCD symptoms or disorder (n=1)
Injury (n=76)	0.78 (0.67‐0.88)	0.84 (0.77‐0.90)	10,470	Self harm (n=8), miscellaneous psychiatric symptoms (n=3), and stress-related symptoms or disorder (n=2)
Psychotic symptoms or disorder (n=50)	0.76 (0.63‐0.86)	0.76 (0.66‐0.85)	6074	Miscellaneous psychiatric symptoms (n=5), neurocognitive symptoms (n=2), and impulsive behavior (n=1)
Stress-related symptoms or disorder (n=11)	0.73 (0.43‐0.90)	0.57 (0.32‐0.77)	480	Stressor symptoms (n=2) and anxiety symptoms (n=1)
Anxiety disorder (n=14)	0.71 (0.45‐0.88)	0.71 (0.50‐0.90)	683	Anxiety symptoms (n=1), social situation (n=1), and somatization symptoms (n=1)
Suicidal symptoms (n=12)	0.67 (0.39‐0.86)	0.73 (0.46‐0.92)	6167	Self-harm (n=3) and psychotic symptoms or disorder (n=1)
Self-harm (n=12)	0.67 (0.39‐0.86)	0.47 (0.23‐0.67)	2126	Abusive behavior (n=3) and suicidal symptoms (n=1)
Anxiety symptoms (n=22)	0.64 (0.43‐0.80)	0.54 (0.36‐0.69)	7481	Stress-related symptoms or disorder (n=2), sensory disturbances (n=2), and anxiety disorder (n=2)
Neurocognitive symptoms (n=74)	0.61 (0.49‐0.71)	0.61 (0.50‐0.69)	1802	Neurocognitive disorders (n=10), miscellaneous psychiatric symptoms (n=8), and ADHD spectrum (n=6)
Aggressive symptoms (n=24)	0.58 (0.40‐0.76)	0.58 (0.40‐0.74)	4275	Anxiety symptoms (n=4), mood symptoms (n=4), and miscellaneous psychiatric symptoms (n=2)
Depressive symptoms (n=39)	0.56 (0.41‐0.71)	0.68 (0.54‐0.80)	6381	Mood symptoms (n=5), miscellaneous psychiatric symptoms (n=3), and unipolar depressive disorder (n=2)
Pharm symptoms (n=7)	0.43 (0.16‐0.75)	0.33 (0‐0.59)	699	Sensory disturbances (n=2), psych ADE^d^ (n=1), and miscellaneous psychiatric symptoms (n=1)
Bipolar spectrum (n=36)	0.42 (0.27‐0.58)	0.59 (0.40‐0.74)	2290	Mood symptoms (n=18), psychotic symptoms or disorder (n=2), and miscellaneous psychiatric symptoms (n=1)
Miscellaneous psychiatric symptoms (n=156)	0.29 (0.22‐0.36)	0.39 (0.30‐0.46)	9554	Neurocognitive symptoms (n=17), antisocial behavior (n=10), and mood symptoms (n=10)
Suicidal behavioral (n=12)	0.25 (0.09‐0.53)	0.38 (0‐0.67)	1164	Injury (n=3), miscellaneous psychiatric symptoms (n=1), and overdose (n=1)
Antisocial behavior (n=10)	0.20 (0.06‐0.51)	0.17 (0‐0.37)	1666	Personality disorder (n=3), aggressive symptoms (n=2), and miscellaneous psychiatric symptoms (n=2)
Sensory disturbances (n=6)	0.17 (0.03‐0.56)	0.09 (0‐0.27)	387	Psychotic symptoms or disorder (n=3) and miscellaneous psychiatric symptoms (n=2)
Stressor symptoms (n=5)	0 (0‐0.43)	0 (0‐0)	34	Sensory disturbances (n=2), personality disorder (n=2), and miscellaneous psychiatric symptoms (n=1)
Psych ADE (n=11)	0 (0‐0.26)	0 (0‐0)	151	Neurocognitive symptoms (n=6) and pharm symptoms (n=5)

a Recall indicates the proportion of terms in a clinician-coded category that were classified by the model as belonging to that category.

b ADHD: attention deficit hyperactive disorder.

c OCD: obsessive compulsive disorder.

d psych ADE: psychiatric adverse drugs events.

The model exhibited the best classification performance for categories of: “living situation” (*F*_1_-score=1, n=11 terms), “substance use related symptoms and disorder” (*F*_1_-score=0.94, n=90 terms), “eating disorder or symptoms” (*F*_1_-score=0.95, n=16 terms), “OCD symptoms or disorder” (*F*_1_-score=0.87, n=10 terms), and “sleep wake symptoms or disorder” (*F*_1_-score=0.86, n=37 terms). Conversely, the model performed poorly on “miscellaneous psychiatric symptoms” (*F*_1_-score=0.39, n=156 terms), “antisocial behavior” (*F*_1_-score=0.17, n=10 terms), “sensory disturbances” (*F*_1_-score=0.09, n=10 terms), “psychiatric adverse drug events” (*F*_1_-score=0, n=11 terms), and “stressor symptoms” (*F*_1_-score=0, n=5 terms).

The most mislabeled mental health terms included “psychiatric adverse drug events” as “neurocognitive symptoms” (n=6 misclassifications) or “pharmacological symptoms” (n=5 misclassifications). The model also commonly mislabeled terms in “miscellaneous psychiatric symptoms.” There were 111 terms in the “miscellaneous psychiatric symptoms” category that were misclassified across 28 of 41 other categories (Multimedia Appendix 3).

### Physical Health

Physical health terms (n=3707) were classified into 19 categories with κ of 0.69 (95% CI 0.67-0.70), precision of 0.76 (95% CI 0.74‐0.77), recall of 0.71 (95% CI 0.70‐0.73), and *F*_1_-score of 0.72 (95% CI 0.70‐0.73). Table 3 includes category-wise recall, *F*_1_-score, and a set of the most frequent categories into which terms from the true category were misclassified (Multimedia Appendix 3).

**Table 3.** Physical health term categories: recall, *F*_1_-score, total mentions in the dataset, and most common misclassification (in descending order of recall).

Term category (n)	Recall (95% CI)^a^	*F*_1_-score (95% CI)	Total mentions in dataset (thousands)	Most frequent misclassifications (n)
Oncological conditions (n=45)	0.91 (0.79‐0.96)	0.61 (0.51‐0.70)	4549	Autoimmune and inflammatory conditions (n=1), gastrointestinal symptoms (n=1), and other physical symptoms and conditions (n=1)
Sensory problems (n=41)	0.90 (0.78‐0.96)	0.35 (0.27‐0.43)	3113	Neurological symptoms (n=4)
Cardiovascular symptoms (n=401)	0.88 (0.85‐0.91)	0.88 (0.85‐0.90)	30,930	Other physical symptoms and conditions (n=14), neurological symptoms (n=9), and respiratory symptoms (n=8)
Respiratory symptoms (n=139)	0.84 (0.77‐0.89)	0.72 (0.66‐0.77)	27,775	Sensory problems (n=6), gastrointestinal symptoms (n=5), and other physical symptoms and conditions (n=4)
Infectious symptoms (n=145)	0.84 (0.77‐0.89)	0.63 (0.57‐0.68)	15,079	Hepatobiliary conditions (n=7), sensory problems (n=3), and skin and soft tissue disorders (n=3)
Metabolic disorders (n=63)	0.84 (0.73‐0.91)	0.68 (0.59‐0.76)	3136	Hepatobiliary conditions (n=7), endocrine symptoms (n=1), and other physical symptoms and conditions (n=1)
Hematological symptoms (n=122)	0.83 (0.75‐0.89)	0.81 (0.75‐0.86)	6321	Oncological conditions (n=11), gastrointestinal symptoms (n=3), and hepatobiliary conditions (n=3)
Neurological symptoms (n=413)	0.82 (0.78‐0.85)	0.79 (0.76‐0.82)	22,540	Sensory problems (n=38), other physical symptoms and conditions (n=8), and infectious symptoms (n=5)
Gastrointestinal symptoms (n=279)	0.81 (0.76‐0.85)	0.77 (0.72‐0.81)	24,878	Hepatobiliary conditions (n=18), autoimmune and inflammatory conditions (n=10), and infectious symptoms (n=9)
Skin and soft tissue disorders (n=314)	0.78 (0.73‐0.82)	0.80 (0.76‐0.83)	15,212	Infectious symptoms (n=26), other physical symptoms and conditions (n=13), and gastrointestinal symptoms (n=9)
Genitourinary symptoms (n=201)	0.77 (0.71‐0.82)	0.81 (0.76‐0.85)	8571	Gastrointestinal symptoms (n=12), infectious symptoms (n=11), and other physical symptoms and conditions (n=7)
Renal disorders (n=52)	0.75 (0.62‐0.85)	0.76 (0.65‐0.84)	2221	Infectious symptoms (n=5), genitourinary symptoms (n=4), and cardiovascular symptoms (n=3)
Endocrine symptoms (n=98)	0.67 (0.58‐0.76)	0.71 (0.63‐0.78)	4942	Metabolic disorders (n=16), sensory problems (n=4), and autoimmune and inflammatory conditions (n=3)
Musculoskeletal symptoms (n=480)	0.67 (0.63‐0.71)	0.79 (0.75‐0.82)	21,785	Other physical symptoms and conditions (n=62), neurological symptoms (n=39), and autoimmune and inflammatory conditions (n=13)
Pain symptoms (n=59)	0.59 (0.47‐0.71)	0.61 (0.51‐0.71)	18,045	Other physical symptoms and conditions (n=6), neurological symptoms (n=5), and gastrointestinal symptoms (n=4)
Autoimmune and inflammatory conditions (n=68)	0.54 (0.43‐0.66)	0.50 (0.40‐0.60)	6234	Infectious symptoms (n=9), other physical symptoms and conditions (n=9), and skin and soft tissue disorders (n=4)
Hepatobiliary conditions (n=54)	0.54 (0.41‐0.66)	0.45 (0.33‐0.56)	1970	Gastrointestinal symptoms (n=11), cardiovascular symptoms (n=4), and other physical symptoms and conditions (n=3)
Other physical symptoms and conditions (n=559)	0.47 (0.42‐0.51)	0.54 (0.50‐0.58)	31,151	Sensory problems (n=68), neurological symptoms (n=39), and skin and soft tissue disorders (n=29)
Respiratory disorders (n=173)	0.40 (0.33‐0.47)	0.55 (0.48‐0.63)	7120	Respiratory symptoms (n=50), infectious symptoms (n=28), and other physical symptoms and conditions (n=10)

a Recall indicates the proportion of terms in a clinician-coded category that were classified by the model as belonging to that category.

The model exhibited the best classification performance for categories of: “cardiovascular symptoms” (n=401 terms), “hematological symptoms” (n=122 terms), and “genitourinary symptoms” (n=201 terms), with recall and *F*_1_-score values >0.80. Conversely, the model performed poorly on “sensory problems” (*F*_1_-score=0.35, n=41 terms), “hepatobiliary conditions” (*F*_1_-score=0.45, n=54 terms), and “other physical symptoms and conditions” (*F*_1_-score=0.54, n=559 terms).

The model commonly predicted the category “sensory problems” in terms of the categories “other physical symptoms and conditions” (n=68 misclassifications) and “neurological symptoms” (n=38 misclassifications). The model also commonly mislabeled “other physical symptoms and conditions.” There were 299 “other physical symptoms and conditions” terms that were misclassified across 18 other categories (Multimedia Appendix 3).

## Discussion

### Principal Findings

We investigated a GPT-4’s ability to replicate clinical judgment when classifying EHR terms from a dataset of mental health patients into interpretable clinical categories. A recent review of NLP studies found the agreement of human coding of EHR data to range from 0.72 to 0.94 (Cohen κ) [28]. Based on this benchmark, GPT-4 showcases human-like agreement with clinical experts when classifying EHR terms as either mental or physical health. Yet, GPT-4’s classification performance varied widely across mental health and physical health categories and had high error rates for certain categories (eg, “sensory problems” and “stressor symptoms”). Misclassifications highlighted GPT-4’s biases, such as the tendency for broad categories (eg, “other physical symptoms and conditions”) to be underselected. Instead, terms from these categories were allocated to more specific categories (eg, “cutting” was allocated to “injury” instead of “self-harm”).

Nevertheless, GPT-4 was able to rapidly transform a feature set of 4553 individual EHR terms into 61 clinically valid groups which can be readily implemented into prediction models. State-of-the-art LLMs have already been used alongside traditional NLP methods, such as named entity recognition, text clustering, and supervised machine learning models trained on text data [29-31]. Additionally, LLMs can explain categorization decisions, providing valuable insights for end users of integrated clinical tools.

### Limitations

LLMs occasionally “hallucinate”, generating outputs that are off-task, nonsensical, or contradictory. Although we prompted the model to validate the output and correct for hallucinations, as the creativity and complexity of tasks increase so does the risk of aberrant outputs [32]. Moreover, recent studies have found that LLM performance on certain clinical tasks can substantially improve when given 1 or multiple examples for codes, a process known as “few-shot” learning [33,34]. In contrast, our study used “zero-shot” learning, where GPT-4 was asked to classify clinical terms without being provided with any specific examples or definitions for the coding system. This method was chosen to assess the model’s baseline classification performance, without introducing any more task-specific bias. However, we recognize that because the coding system was developed by only 2 clinicians, bias may be introduced due to their unique sets of clinical experiences, institutional practices, and personal preferences. The LLM may be biased as well. An ad hoc analysis indicated a tendency for the model to underuse “other” categories (eg, “other physical symptoms and conditions” and “miscellaneous psychiatric symptoms”) relative to clinician coders (Multimedia Appendix 3). Nonetheless, we acknowledge that many clinical terms in EHR are inherently ambiguous and may be classified under multiple categories depending on the context. Without knowing the sample is among people hospitalized with a mental health disturbance, it is not necessarily a misclassification for GPT-4 to label “gunshot wound” as a physical injury and not an indicator of suicide. The task of assigning a single, mutually exclusive label may limit one’s ability to capture the full complexity of the clinical term. While this study provides a preliminary framework for exploring the feasibility of using LLMs for unstructured EHR classification, future research should aim to involve a varied set of coding methods, classification approaches (eg, multi-label classification), and a larger cohort of clinician-coders to enhance generalizability. Finally, we note that several categories in the mental health domain had too few terms (<5) to yield stable estimates of agreement and were removed from the analysis.

### Implications

The accuracy of clinical term classification is essential for downstream predictive models that rely on structured data, as inaccuracies can propagate through the model pipeline. Understanding the sensitivity of these models to variations in input labels is key, especially when distinguishing between random errors and systematic misclassifications. Systematic errors, where specific categories are consistently mislabeled, may significantly affect the robustness of models trained on such data, potentially more so than a random error (ie, noise) [35-37]. Moreover, the assumption that accurate categorization of clinical terms is a necessary intermediate step is worth reconsidering. As LLMs advance, there is potential for these models to bypass the traditional 2-stage process and make direct predictions from unstructured text [30]. Future research is needed to determine whether bypassing the intermediate categorization step entirely might enhance or hinder model performance, depending on the specific clinical application.

### Conclusion

As LLMs continue to advance, the time and human resources required to distill a large corpus of EHR terms into clinically meaningful groups can be greatly reduced. LLMs have the potential to be integrated into EHR systems to create text-based features for prediction models in real time. This study found that a state-of-the-art LLM achieved high agreement with classifications of experienced clinicians across terms from numerous physical and mental health categories.

## Supplementary material

10.2196/65454 Multimedia Appendix 1 Code and prompt design.

10.2196/65454 Multimedia Appendix 2 Classification performance metrics output.

10.2196/65454 Multimedia Appendix 3 Analysis of misclassifications.

## Abbreviations

ED: emergency department
EHR: electronic health record
LLM: large language model
NLP: natural language processing
