# Supplementary material for: Classifying Unstructured Text in Electronic Health Records for Mental Health Prediction Models: Large Language Model Evaluation Study
Source: JMIR Med Inform. 2025 Jan 21;13:e65454. doi: 10.2196/65454 (PMC11884378; doi:10.2196/65454)
Supplement: Multimedia Appendix 1 [file medinform-v13-e65454-s001.docx]

*Multimedia Appendix 1 – Code and Prompt Design*

Python code for task to categorize term as “physical health” or “mental health”.

from pydantic import BaseModel

import instructor

from google.colab import userdata

import json

import openai

import os  # Assuming 'os' module is used for os.chdir

# my directory, remember to mount drive

working_directory = "/content/drive/MyDrive/EHR GPT Coding Project/"

os.chdir(working_directory)  # set directory

# Open AI API key stored in userdata.

openai.api_key = userdata.get('key')

# Apply the patch to the OpenAI client

# enables response_model keyword

client = instructor.patch(openai.OpenAI(api_key=userdata.get('key')))

def classify(content):

    """Perform single-label classification on the input text."""

    categories = ["MH", "PH"]

    prompt = f"We extracted clinical terms from the Electronic Health Records from people hospitalized for mental health related problems. You are a Electronic Health Record term classification machine. Classify the electronic medical record text I give you as 'MH' if you think it is a mental health term or 'PH' if you think it is a physical health term. Only return text that matches exactly with the listed categories. Do not return any text which isn't 'MH' or 'PH'.\n\nText: {content}\nCategory:"

    response = openai.chat.completions.create(

        model="gpt-4-turbo-preview",

        messages=[{"role": "system", "content": "You can only respond with one of the following: 'MH', 'PH'. When you don't know, make a guess."},

                  {"role": "user", "content": prompt}],

        seed=3535,

        temperature=0,

        max_tokens=60,

        top_p=1,

        frequency_penalty=0,

        presence_penalty=0,

        n=1,

        stop="\n"

    )

    predicted_category = response.choices[0].message.content

    if predicted_category in categories:

        return predicted_category

    else:

        verif_prompt = f"Please classify {predicted_category} into one of the following categories: 'MH', 'PH'. When you don't know, make a guess. Category: "

        verified_response = openai.chat.completions.create(

            model="gpt-4-turbo-preview",

            messages=[{"role": "system", "content": "You can only respond with one of the following terms: 'MH', 'PH'."},

                      {"role": "user", "content": verif_prompt}],

            seed=3535,

            temperature=0,

            max_tokens=60)

        return verified_response.choices[0].message.content

Prompt design: task to categorize mental health term into mental health categories.

import openai

import enum

from pydantic import BaseModel

import instructor

from google.colab import userdata

import json

import os

working_directory = "/content/drive/MyDrive/EHR GPT Coding Project/" # my directory, remember to mount drive

os.chdir(working_directory) # set directory

openai.api_key = userdata.get(key') # Open AI API key

# Apply the patch to the OpenAI client

# enables response_model keyword

client = instructor.patch(openai.OpenAI(api_key=userdata.get('key')))

def classify(content):

    """Perform single-label classification on the input text."""

    categories = [

"abusive_behavior",

"adhd_spectrum",

"aggressive_symptoms",

"antisocial_behavior",

"anxiety_disorder",

"anxiety_symptoms",

"autism_spectrum_disorder",

"bipolar_spectrum",

"depressive_symptoms",

"eating_disorder_or_symptoms",

"gender_identity_disorder",

"homeless",

"impulsive_behavior",

"injury",

"lives_alone",

"living_situation",

"maladaptive_relations",

"medication_use",

"miscellaneous_psychiatric_symptoms",

"mixed_anxiety_depression",

"mood_disorder",

"mood_symptoms",

"neurocognitive_disorders",

"neurocognitive_symptoms",

"ocd_symptoms_or_disorder",

"overdose",

"personality_disorder",

"pharm_symptoms",

"psych_ade",

"psychotic_symptoms_or_disorder",

"self_harm",

"sensory_disturbances",

"sleep_wake_symptoms_or_disorder",

"social_situation",

"somatization_symptoms",

"stress_related_symptoms_or_disorder",

"stressor_symptoms",

"substance_related_symptoms_or_disorder",

"suicidal_behavioral",

"suicidal_symptoms",

"suicide",

"unipolar_depressive_disorder"

    ]

#

    prompt = f"We extracted clinical terms from the Electronic Health Records from people hospitalized for mental health related problems. You are a Electronic Health Record term classification machine. Classify the electronic medical record term I give you into one of these categories. Only return text that matches exactly with the listed categories. Here are the possible categories: {', '.join(categories)}.\n\nText: {content}\nCategory:"

    response = openai.chat.completions.create(

        model="gpt-4-turbo-2024-04-09",

        messages=[{"role": "system", "content": f"You can only respond with one of the following terms: {', '.join(categories)}. When you don't know, make a guess."},

                  {"role": "user", "content": prompt}],

        seed=3535,

        temperature=0,

        max_tokens=60,

        top_p=1,

        frequency_penalty=0,

        presence_penalty=0,

        n=1,

        stop="\n"

    )

    predicted_category = response.choices[0].message.content

    token_count1 = response.usage.total_tokens

    if predicted_category in categories:

        return predicted_category #, token_count1

    else:

       verif_prompt = f"Please classify {predicted_category} into one of the following categories: {', '.join(categories)}. When you don't know, make a guess. Category:"

       verified_response = openai.chat.completions.create(

                model="gpt-4-turbo-2024-04-09",

                messages=[{"role": "system", "content": f"You can only respond with one of the following terms: {', '.join(categories)}."},

                          {"role": "user", "content": verif_prompt}],

                seed=3535,

                temperature=0,

                max_tokens=60)

       return verified_response.choices[0].message.content, predicted_category, "*"

Python code for task to categorize physical health term into physical health categories.

import openai

import enum

from pydantic import BaseModel

import instructor

from google.colab import userdata

import json

import os

working_directory = "/content/drive/MyDrive/EHR GPT Coding Project/" # my directory, remember to mount drive

os.chdir(working_directory) # set directory

openai.api_key = userdata.get(key') # Open AI API key

# Apply the patch to the OpenAI client

# enables response_model keyword

client = instructor.patch(openai.OpenAI(api_key=userdata.get('key')))

def classify(content):

    """Perform single-label classification on the input text."""

    categories = [

"autoimmune_and_inflammatory_conditions",

"cardiovascular_symptoms",

"endocrine_symptoms",

"gastrointestinal_symptoms",

"genitourinary_symptoms",

"hematological_symptoms",

"hepatobiliary_conditions",

"infectious_symptoms",

"metabolic_disorders",

"musculoskeletal_symptoms",

"neurological_symptoms",

"oncological_conditions",

"other_physical_symptoms_and_conditions",

"pain_symptoms",

"renal_disorders",

"respiratory_disorders",

"respiratory_symptoms",

"sensory_problems",

"skin_and_soft_tissue_disorders"

    ]

#

    prompt = f"We extracted clinical terms from the Electronic Health Records from people hospitalized for mental health related problems. You are a Electronic Health Record term classification machine. Classify the electronic medical record term I give you into one of these categories. Only return text that matches exactly with the listed categories. Here are the possible categories: {', '.join(categories)}.\n\nText: {content}\nCategory:"

    response = openai.chat.completions.create(

        model="gpt-4-turbo-2024-04-09",

        messages=[{"role": "system", "content": f"You can only respond with one of the following terms: {', '.join(categories)}. When you don't know, make a guess."},

                  {"role": "user", "content": prompt}],

        seed=3535,

        temperature=0,

        max_tokens=60,

        top_p=1,

        frequency_penalty=0,

        presence_penalty=0,

        n=1,

        stop="\n"

    )

    predicted_category = response.choices[0].message.content

    token_count1 = response.usage.total_tokens

    if predicted_category in categories:

        return predicted_category #, token_count1

    else:

       verif_prompt = f"Please classify {predicted_category} into one of the following categories: {', '.join(categories)}. When you don't know, make a guess. Category:"

       verified_response = openai.chat.completions.create(

                model="gpt-4-turbo-2024-04-09",

                messages=[{"role": "system", "content": f"You can only respond with one of the following terms: {', '.join(categories)}."},

                          {"role": "user", "content": verif_prompt}],

                seed=3535,

                temperature=0,

                max_tokens=60)

       return verified_response.choices[0].message.content, predicted_category, "*"

Python code to run the prompt across respective dataset of terms:

import pandas as pd # pandas

terms_df = pd.read_csv("terms.csv").fillna(0)

columns_to_drop = ["cat"]

# Drop the specified columns

terms_df = terms_df.drop(columns=columns_to_drop)

terms_df = terms_df

terms_df = pd.DataFrame(terms_df)

 #13 mins 21 seconds for 1000 terms

# Define your classify function and enum

# (Include all the required imports and definitions here)

# Function to classify strings in a DataFrame

def classify_strings(df, column_name):

    # Create a new column for storing predicted labels

    df['pred_cat'] = ''

    # Iterate over each row

    for index, row in df.iterrows():

        # Get the string from the specified column

        string_to_classify = row[column_name]

        #print("Input:", string_to_classify)

        # Perform classification

        prediction = classify(string_to_classify)

        #print("Output:", prediction)

        # Get the predicted label from the response

        predicted_label = prediction

        # Store the predicted label in the new column

        df.at[index, 'pred_cat'] = predicted_label

    return df

# Call the function to classify the strings

df_with_predictions = classify_strings(terms_df, 'term')

df_with_predictions
